# Investigating the Structural Evolution and Catalytic Activity of *c*-Co/Co_3_Mo Electrocatalysts for Alkaline Hydrogen Evolution Reaction

**DOI:** 10.3390/molecules28196986

**Published:** 2023-10-09

**Authors:** Long Chen, Li-Wen Jiang, Jian-Jun Wang

**Affiliations:** 1 State Key Laboratory of Crystal Materials, Shandong University, Jinan 250100, China; 202232804@mail.sdu.edu.cn (L.C.); 201812674@mail.sdu.edu.cn (L.-W.J.); 2 Shenzhen Research Institute, Shandong University, Shenzhen 518057, China

**Keywords:** transition metal alloys, electrocatalysts, structural stability, alkaline hydrogen evolution reaction (HER), *c*-Co/Co_3_Mo electrocatalyst

## Abstract

Transition metal alloys have emerged as promising electrocatalysts due to their ability to modulate key parameters, such as d-band electron filling, Fermi level energy, and interatomic spacing, thereby influencing their affinity towards reaction intermediates. However, the structural stability of alloy electrocatalysts during the alkaline hydrogen evolution reaction (HER) remains a subject of debate. In this study, we systematically investigated the structural evolution and catalytic activity of the *c*-Co/Co_3_Mo electrocatalyst under alkaline HER conditions. Our findings reveal that the Co_3_Mo alloy and H_0.9_MoO_3_ exhibit instability during alkaline HER, leading to the breakdown of the crystal structure. As a result, the cubic phase *c*-Co undergoes a conversion to the hexagonal phase *h*-Co, which exhibits strong catalytic activity. Additionally, we identified hexagonal phase Co(OH)_2_ as an intermediate product of this conversion process. Furthermore, we explored the readsorption and surface coordination of the Mo element, which contribute to the enhanced catalytic activity of the *c*-Co/Co_3_Mo catalyst in alkaline HER. This work provides valuable insights into the dynamic behavior of alloy-based electrocatalysts, shedding light on their structural stability and catalytic activity during electrochemical reduction processes.

## 1. Introduction

Reducing CO_2_ emissions has emerged as a global development goal, driving the search for clean and low-carbon renewable energy sources [1,2,3,4,5,6]. Among these, hydrogen energy has garnered significant attention as a clean and zero-carbon emission alternative [7,8,9,10]. Electrochemical water splitting, powered by electricity from renewable sources, presents a promising technology for producing “green hydrogen” [11,12,13,14]. However, the widespread adoption of this technology is hindered by the low efficiency of hydrolysis, particularly in alkaline water electrolysis cells, due to the higher operating overpotentials of the hydrogen evolution reaction (HER) [15,16]. Unlike the direct formation of hydrogen intermediates (H*) from proton reduction (H^+^) in acidic media, the alkaline HER process involves the splitting of water molecules to generate hydrogen intermediates, resulting in increased energy consumption and reduced efficiency [17,18]. Therefore, the development and design of highly efficient and stable electrocatalysts for alkaline HER is crucial for enhancing the efficiency and reducing the cost of alkaline water electrolysis cells.

To date, noble metal-based materials have been considered as the benchmark electrocatalysts for the hydrogen evolution reaction (HER). However, their high cost limits their widespread application [17,19,20,21]. In order to replace noble metal-based electrocatalysts, cost-effective alloy electrocatalysts composed of transition metals have been developed for alkaline HER [22,23,24]. The alloying of transition metals allows for the tuning of d-band electron filling, Fermi level energy, and interatomic spacing, all of which can impact the affinity of the alloy electrocatalysts towards reaction intermediates [25,26,27,28]. However, the structural stability of alloy electrocatalysts, particularly those containing Mo elements, during the alkaline HER process has been a subject of controversy. For instance, in Ni_4_Mo alloy, Mo was found to dissolve and form MoO_4_^2−^ during alkaline HER, with the surface-adsorbed Mo_2_O_7_^2−^ promoting the HER activity of metal Ni [29]. This can be attributed to the high affinity between Mo and oxygen, facilitating the bonding of Mo atoms with hydroxyl groups in the alkaline electrolyte [30]. Similarly, in IrMo alloy, it has been confirmed that the surface of IrMo exhibits easier adsorption of OH compared to pure Ir [30]. Additionally, for Co-based electrocatalysts, in situ cobalt metal formed by electrochemical reduction has been identified as the true catalytically active species for HER [31,32]. Therefore, it is necessary to explore the structural stability of Co_3_Mo alloys, despite their widely reported and studied catalytic activity as excellent electrocatalysts [33,34]. In this study, we systematically investigated the formation of hexagonal cobalt (*h*-Co) from hexagonal Co(OH)_2_ as an intermediate species, along with the readsorption and surface coordination of Mo elements, leading to enhanced catalytic activity for alkaline HER.

## 2. Results and Discussion

The *c*-Co/Co_3_Mo electrocatalyst on carbon cloth was synthesized by annealing the Co(OH)_2_/CoMoO_4_ precursor, which was obtained through hydrothermal treatment of α-Co(OH)_2_ nanosheet arrays in a NaMoO_4_ solution. Carbon cloth was chosen as a binder-free substrate for supporting the catalyst due to its high conductivity, three-dimensional (3D) microstructure, and excellent chemical/mechanical stability [35]. The crystal structure and composition of the *c*-Co/Co_3_Mo electrocatalysts can be optimized by adjusting the annealing and hydrothermal conditions of the precursors [36]. Similarly, the crystal structure and composition of the Co(OH)_2_/CoMoO_4_ precursor before annealing were controlled by adjusting the hydrothermal temperature, time, and concentration. Experimental results showed that increasing the temperature, concentration, and time of the hydrothermal reaction reduced the content of Co(OH)_2_ and increased the content of CoMoO_4_ in the precursor. The XRD patterns (Figures S1–S3) of the precursor indicated the continued dominance of the α-Co(OH)_2_ phase. Additionally, weak peaks corresponding to CoMoO_4_ were observed at approximately 14°, 20°, 37°, 54°, and 65°. After annealing at 500 °C for 2 h, a series of electrocatalysts with different component ratios were obtained with the presence of *c*-Co and Co_3_Mo confirmed by the weak peaks observed at ~45° and 30° in the XRD pattern (Figure S4) probably due to small crystal size or low crystallinity [34], demonstrating the adjustability of the precursor for achieving optimized electrocatalysts. Varying the annealing temperature revealed that annealing at 400 °C mainly resulted in the formation of CoO, while an annealing temperature of 600 °C favored the formation of Co_3_Mo alloy and reduced the content of H_0.9_MoO_3_ (Figure S4a). The annealing time also influenced the phase composition, with the proportion of H_0.9_MoO_3_ initially increasing and then decreasing as the annealing time was increased from 1 h to 3 h at 500 °C (Figure S4b). However, Co_3_Mo alloy was not observed even after extending the annealing time to 3 h, highlighting the critical role of annealing temperature. SEM images confirmed the preservation of the nanosheet arrays of the target electrocatalysts after adjusting the temperature and time of the hydrothermal reaction and annealing process. Subsequently, the electrocatalysts’ HER performance in an alkaline electrolyte was evaluated to select the optimal electrocatalyst. The electrocatalyst obtained by annealing at 500 °C for 2 h exhibited the lowest overpotential at the same current density and was chosen as the targeted electrocatalyst.

**Figure 1:**
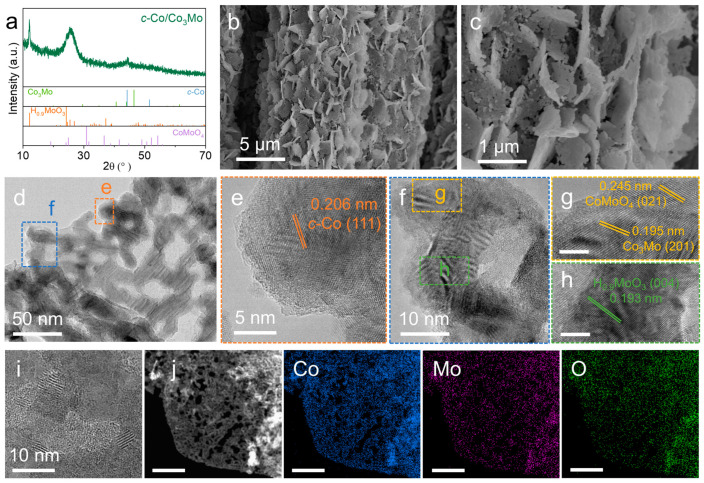
(**a**) XRD pattern, (**b**,**c**) SEM images, (**d**) TEM image, (**e**–**i**) HRTEM images, and (**j**) the corresponding EDX elemental mapping images of *c*-Co/Co_3_Mo. Scale bars: 3 nm (**g**), 3 nm (**h**), and 200 nm (**j**).

The X-ray diffraction (XRD) pattern exhibited two strong diffraction peaks at 12.2° and 44.2°, which can be attributed to the (200) facet of monoclinic H_0.9_MoO_3_ (PDF#53-1024) and the (111) facet of cubic Co (*c*-Co, PDF#15-0806), respectively, confirming the formation of *c*-Co and H_0.9_MoO_3_. The scanning electron microscopy (SEM) image in Figure 1b shows that the *c*-Co/Co_3_Mo nanosheet arrays uniformly cover the surface of the carbon cloth. Upon further magnification of the SEM image (Figure 1c), it can be observed that the porous nanosheet is composed of smaller-sized nanoparticles. To gain further insight into the crystal structure and nanomorphology of the catalyst, transmission electron microscopy (TEM) was employed. The TEM image in Figure 1d reveals that the nanoparticles have sizes ranging from 20 to 30 nm. The high-resolution TEM (HRTEM) image in Figure 1e clearly shows a lattice d-spacing of 0.206 nm, corresponding to the (111) facet of the *c*-Co phase. In Figure 1g, the lattice d-spacings of 0.245 nm and 0.195 nm can be assigned to the (021) facet of CoMoO_4_ and the (201) facet of Co_3_Mo, respectively. Furthermore, the lattice d-spacing of 0.193 nm corresponds to the (004) facet of H_0.9_MoO_3_ (Figure 1h), and the larger layer spacing of approximately 0.72 nm, attributed to the (200) facet of H_0.9_MoO_3_, can be observed due to its two-dimensional layered structure. These HRTEM images indicate that during the thermal reduction process, Co(OH)_2_ and only a portion of CoMoO_4_ in the Co(OH)_2_/CoMoO_4_ precursor are transformed into *c*-Co and Co_3_Mo alloy, respectively. Additionally, a portion of CoMoO_4_ in the precursor is reduced to H_0.9_MoO_3_. These results demonstrate that the synthesized electrocatalyst consists of multiple crystalline phases, including *c*-Co, Co_3_Mo, H_0.9_MoO_3_, and CoMoO_4_. Moreover, the scanning TEM (STEM) image and corresponding energy dispersive X-ray spectroscopy (EDX) elemental mapping images (Figure 1j) confirm that Co, Mo, and O elements are uniformly distributed in the porous nanosheets with the percentages of Co, Mo, and O being 61.76%, 13.94%, and 24.30% (Table S1), respectively.

**Figure 2:**
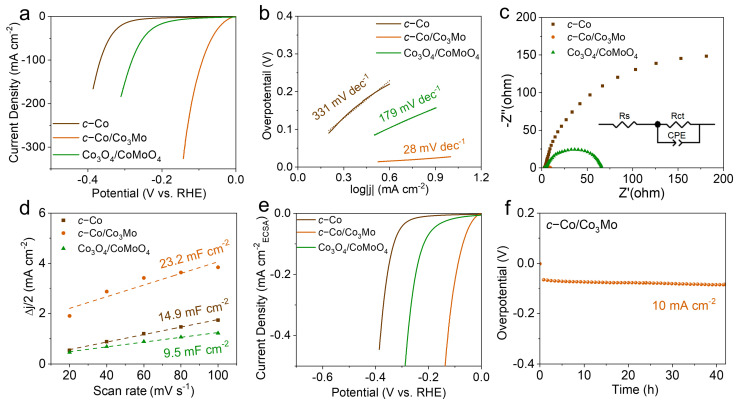
(**a**) LSV curves, (**b**) Tafel slopes, (**c**) Nyquist plots, (**d**) C_dl_ values, and (**e**) ECSAnormalized LSV curves of *c*-Co, *c*-Co/Co_3_Mo, and Co_3_O_4_/CoMoO_4_. (**f**) Chronopotentiometry curve of *c*-Co/Co_3_Mo at a constant current density of 10 mA cm^−2^.

To assess the catalytic activity and stability of the *c*-Co/Co_3_Mo electrocatalyst, a series of electrochemical tests were conducted in alkaline electrolytes. For comparison, the hydrogen evolution reaction (HER) performance of *c*-Co and Co_3_O_4_/CoMoO_4_ catalysts was also evaluated under the same conditions. The linear sweep voltammetry (LSV) curves shown in Figure 2a highlight the remarkable HER performance of the *c*-Co/Co_3_Mo catalyst, as it achieved a current density of 10 mA cm^−2^ with an overpotential of only 28 mV, much lower than that of *c*-Co (280 mV) and Co_3_O_4_/CoMoO_4_ (171 mV). This performance surpasses that of some state-of-the-art Pt-free HER catalysts (Table S2). Furthermore, the electrocatalytic activity of *c*-Co/Co_3_Mo outperformed that of the control sample, the 20% Pt/C electrode, particularly in the high potential range (Figure S12). Meanwhile, it is worth noting that the Tafel slope of *c*-Co/Co_3_Mo was measured to be 28 mV dec^−1^, which is significantly lower than those of the control samples of *c*-Co (331 mV dec^−1^) and Co_3_O_4_/CoMoO_4_ (179 mV dec^−1^). This observation suggests that the *c*-Co/Co_3_Mo electrocatalyst demonstrates faster kinetics, which can be attributed to the Volmer–Tafel mechanism. It is interesting to note that this mechanism differs from the Volmer–Heyrovsky mechanism observed in *c*-Co and Co_3_O_4_/CoMoO_4_ [37,38]. Furthermore, the high electrocatalytic activity of the *c*-Co/Co_3_Mo catalyst is also evident from the TOF values at different potentials (Table S3), which were found to be higher than that of the Pt/C electrode [39]. This comparison further highlights the favorable performance of the c-Co/Co_3_Mo catalyst in the electrocatalytic HER reaction. Electrochemical impedance spectroscopy (EIS) was employed to investigate the charge transfer behavior at the interface between the electrocatalyst and the electrolyte [40]. The Nyquist plots in Figure 2c reveal that the *c*-Co/Co_3_Mo electrocatalyst exhibited the lowest charge transfer resistance (R_ct_) compared to the control samples of *c*-Co and Co_3_O_4_/CoMoO_4_. This indicates that the *c*-Co/Co_3_Mo catalyst promotes electron transfer at the interface, facilitating the alkaline HER process [41]. Furthermore, the electrochemical surface area (ECSA) values were evaluated to explore the intrinsic activity of the electrocatalysts by the corresponding electrochemical double layer capacitance (C_dl_) derived from cyclic voltammetry (CV) curves at different scan rates in the non-Faradaic region (Figure S13), and the current density after the normalization of the electrochemical surface area (ECSA) was calculated [42]. As shown in Figure 2d, the *c*-Co/Co_3_Mo electrocatalyst exhibited a larger C_dl_ value of 23.2 mF cm^−2^ compared to *c*-Co (14.9 mF cm^−2^) and Co_3_O_4_/CoMoO_4_ (9.5 mF cm^−2^). The larger ECSA suggests that the *c*-Co/Co_3_Mo catalyst exposes more catalytically active sites during the alkaline HER process [43]. The ECSA-normalized LSV curves in Figure 2e further confirm that the *c*-Co/Co_3_Mo electrocatalyst exhibited the highest intrinsic activity among the control samples [44]. In addition to catalytic activity, stability is also crucial for practical applications [16]. The stability of the *c*-Co/Co_3_Mo electrocatalyst was evaluated using chronopotentiometry (CP). As depicted in Figure 2f and Figure S14, the applied potential during continuous operation at current densities of 10 mA cm^−2^ and 100 mA cm^−2^ showed minimal increase even after more than 40 h and 25 h, respectively, suggesting good stability of the *c*-Co/Co_3_Mo catalyst in the alkaline HER process. Finally, we conducted measurements to determine the actual amount of hydrogen generated at a current density of 100 mA cm^−2^. As shown in Figure S15, the measured amount of hydrogen produced closely aligns with the theoretical value, indicating that a Faraday efficiency of ~97% was achieved.

**Figure 3:**
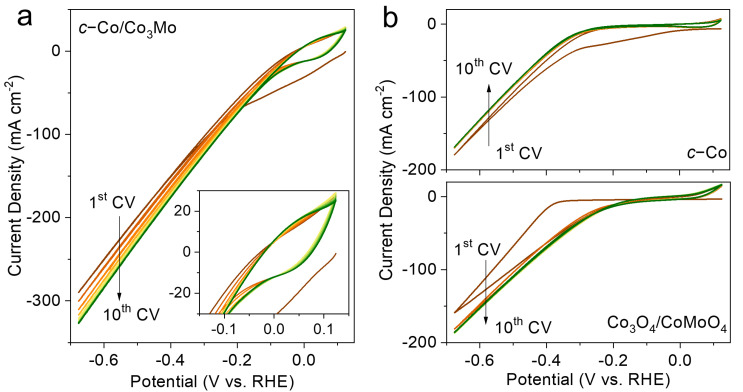
Evolutive CV curves for (**a**) *c*-Co/Co_3_Mo and (**b**) *c*-Co and Co_3_O_4_/CoMoO_4_ from the 1st to the 10th CV in 1 M KOH at 50 mV s^−1^ between 0.124 and −0.676 V (vs. RHE). The inset shows the redox peaks from 0.124 to −0.1 V (vs. RHE).

Furthermore, a series of cyclic voltammetry (CV) curves were recorded for 10 cycles in 1 M KOH at a scan rate of 50 mV s^−1^ between 0.124 and −0.676 V to observe the electrochemical behavior of the electrocatalysts before obtaining stable LSV curves. Interestingly, as shown in Figure 3a, the evolutive CV curves indicate that the *c*-Co/Co_3_Mo electrocatalyst underwent rapid electrochemical activation from the first to the fifth CV in 1 M KOH. As the number of CV cycles increases up to 10, the CV curves become almost perfectly coincident, suggesting stable electrochemical performance. In the inset of Figure 3a, a distinct electrochemical redox peak can be observed from 0.124 to −0.1 V, which may be attributed to the electrochemical transition between cobalt hydroxide and cobalt metal [45]. However, similar electrochemical activation and redox peaks were not observed in the control samples of *c*-Co and Co_3_O_4_/CoMoO_4_. For the control samples, both *c*-Co and Co_3_O_4_/CoMoO_4_ exhibited significant electrochemical instability in the first CV curve and then became stable in the second CV curve (Figure 3b). The difference is that *c*-Co showed a slight decrease in electrochemical performance in the second CV curve, while Co_3_O_4_/CoMoO_4_ showed an enhanced electrochemical performance in the second CV curve.

**Figure 4:**
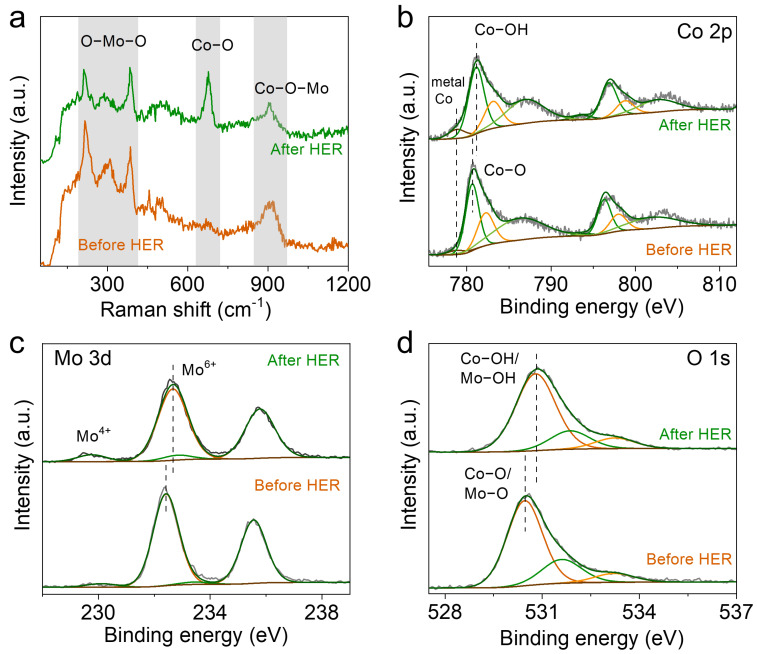
(**a**) Raman spectra and high-resolution XPS spectra of (**b**) Co 2p, (**c**) Mo 3d, and (**d**) O 1s of *c*-Co/Co_3_Mo before and after the HER test.

To investigate the electrochemical activation, we further characterized the crystal and electronic structures of the electrocatalysts. XRD analysis (Figure S16) demonstrated a notable reduction in the diffraction peaks associated with phase H_0.9_MoO_3_ and *c*-Co in the *c*-Co/Co_3_Mo electrocatalyst after the HER test. This indicates a distinct evolution in the crystal structure resulting from electrochemical activation. Furthermore, the enhanced electrochemical activity observed in conjunction with this structural transformation suggests that the presence of H_0.9_MoO_3_ has a negligible effect on the HER activity of the resulting electrocatalyst. In contrast, no significant changes in crystal structure were observed in the control samples of *c*-Co and Co_3_O_4_/CoMoO_4_ (Figure S17). Additionally, Raman spectroscopy was employed to analyze the variation in the lattice vibration of the electrocatalyst before and after the HER test. As shown in Figure 4a, four peaks located at 211, 307, 385, and 906 cm^−1^ can be attributed to the bending vibration of O-Mo-O and the stretching vibration of Co-O-Mo [34,46,47]. After the HER test, the intensity of the vibration modes related to O-Mo-O and Co-O-Mo slightly decreased [48], while a new peak at 673 cm^−1^, corresponding to the stretching vibration of Co-OH, emerged. This suggests that H_0.9_MoO_3_ and a small amount of CoMoO_4_ in the *c*-Co/Co_3_Mo electrocatalyst exhibit poor stability during the alkaline HER process, leading to the formation of cobalt hydroxide.

The electronic structures of the *c*-Co/Co_3_Mo electrocatalyst were further investigated using X-ray photoelectron spectroscopy (XPS). In the Co 2p spectra before the HER test (Figure 4b), two peaks at 778.8 eV and 793.9 eV, corresponding to Co 2p_3/2_ and Co 2p_1/2_ of metallic Co, were observed, along with two peaks at 780.6 eV and 796.3 eV for Co-O [49,50], accompanied by satellite peaks at 786.3 eV and 802.0 eV [51,52]. After the HER test, the intensity of the metallic Co peaks increased, and the two Co-O peaks slightly shifted to higher energies, indicating the presence of Co-OH and confirming the formation of more metallic Co and new cobalt hydroxide [51]. In the Mo 3d region before the HER test (Figure 4c), two peaks at 232.4 eV and 235.6 eV were observed, corresponding to Mo^5+^ in H_0.9_MoO_3_, while the other two peaks at 230.1 eV and 233.5 eV were assigned to Mo^4+^ [53,54]. After the HER test, the two peaks related to Mo^5+^ slightly shifted to higher energies, indicating the presence of Mo^6+^ from MoO_4_^2−^ on the surface. This suggests that the Mo element in the electrocatalyst first dissolves in the alkaline electrolyte, undergoes electrooxidation into MoO_4_^2−^ ions, and then readsorbs onto the electrode surface [29,31]. In the O 1 s region (Figure 4d), three peaks at 530.5 eV, 531.6 eV, and 533.2 eV were observed, corresponding to the Co-O/Mo-O bond, oxygen vacancy, and adsorbed water, respectively [55]. After the HER test, the peak associated with the Co-O/Mo-O bond shifted to higher binding energy, indicating the formation of cobalt hydroxide. These results suggest that during the electrochemical activation, Mo elements are leached from the electrocatalyst into the electrolyte and then readsorbed onto the electrode surface, accompanied by the formation of cobalt hydroxide. Combined with the previous observation of redox peaks (Figure 3a), it is speculated that cobalt hydroxide may undergo further reduction to form metallic cobalt during the alkaline HER process.

**Figure 5:**
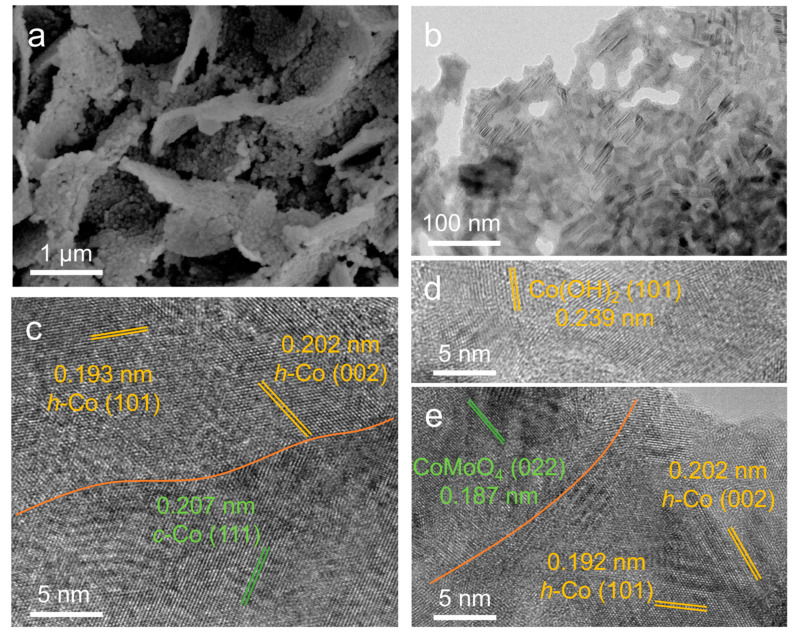
(**a**) SEM image, (**b**) TEM image, and (**c**–**e**) HRTEM images of *c*-Co/Co_3_Mo after the HER test.

To further investigate the structural evolution of the *c*-Co/Co_3_Mo electrocatalyst, electron microscopy techniques were employed to observe the nanomorphology and microscopic crystal structure. The SEM image in Figure 5a shows that the nanomorphology of the *c*-Co/Co_3_Mo electrocatalyst remained unchanged after the HER test. However, TEM images reveal the presence of coated nanoparticles, unlike the individually dispersed nanoparticles observed before the HER test, indicating surface reconstruction in the outer layer of the nanoparticles (Figure 5b). Furthermore, HRTEM images provide insight into the microscopic crystal structure of the *c*-Co/Co_3_Mo electrocatalyst after the HER test. In Figure 5c, the lattice d-spacing of 0.207 nm corresponds to the (111) facet of *c*-Co, while the lattice d-spacings of 0.193 nm and 0.202 nm can be attributed to the (101) and (002) facets of hexagonal Co (*h*-Co). In the outer region of the nanoparticles (Figure 5d), the observed lattice d-spacing of 0.239 nm is assigned to the (101) facets of hexagonal Co(OH)_2_. Additionally, the (022) facet of CoMoO_4_ and the (101) and (002) facets of *h*-Co can also be observed in Figure 5e, confirming the formation of *h*-Co. Notably, the HRTEM images do not show lattice fringes corresponding to H_0.9_MoO_3_ and Co_3_Mo in the *c*-Co/Co_3_Mo electrocatalyst after the HER test, indicating their instability during alkaline HER. STEM images and corresponding EDX elemental mapping images reveal the homogeneous and overlapping distribution of Co, Mo, and O throughout the nanosheets (Figure S18).

To investigate the stability of the Co_3_Mo alloy, we prepared an electrocatalyst consisting of highly crystalline Co_3_Mo alloys by annealing the sample at 120 °C for 8 h in a 0.5 M solution, followed by heating at 600 °C for 2 h. Characterization of the electrocatalyst was performed. The cyclic voltammetry (CV) curves in Figure S19 reveal an electrochemical activation of the Co_3_Mo alloys from the 1st to the 10th CV in a 1 M KOH solution, accompanied by a distinct electrochemical redox peak. X-ray diffraction (XRD) patterns indicated a significant reduction in the intensity of the diffraction peak of the Co_3_Mo alloy, suggesting its instability during the alkaline HER process. Scanning electron microscopy (SEM) images exhibited the characteristic hexagonal nanoplates of Co(OH)_2_, confirming its formation. These findings suggest that the Co_3_Mo alloy undergoes dissolution in the electrolyte during the alkaline HER process, with the dissolved cobalt ions subsequently redepositing on the electrode surface to form Co(OH)_2_. Additionally, the dissolved Mo ions also readhere to the electrode surface, forming coordination polyhedra that regulate the electronic structure of the catalytic site and enhance the catalytic activity during alkaline HER [31]. Therefore, it is reasonable to speculate that both the Co_3_Mo alloy and H_0.9_MoO_3_ are destroyed during the alkaline HER process, resulting in the formation of soluble Mo and Co ions. The Mo ions are then readsorbed on the surface to form coordination polyhedral ions, while the Co ions are redeposited to form Co(OH)_2_. Finally, under a negative electric field, Co(OH)_2_ is further reduced to *h*-Co.

## 3. Materials and Methods

### 3.1. Chemicals

Co(NO_3_)_2_·6H_2_O, hexamethylenetetramine (HMT), NaMoO_4_·2H_2_O, and KOH were obtained from Sinopharm Chemical Reagent Co., Ltd., Shanghai, China. All the chemicals were directly used as received without further purification.

### 3.2. Synthesis of the Co(OH)_2_/CoMoO_4_ Precursor on Carbon Cloth

In accordance with previous reports [31], α-Co(OH)_2_ nanosheet arrays were loaded onto a carbon cloth substrate. To prepare the Co(OH)_2_/CoMoO_4_ precursor (sample-120 °C-8 h-0.5 M), 2.42 g of NaMoO_4_·2H_2_O and 20 mL of deionized water were mixed thoroughly in a 45 mL Teflon autoclave to form a 0.5 M NaMoO_4_ solution. A piece of carbon cloth (2 × 2.5 cm) with α-Co(OH)_2_ was then added to the same autoclave. The autoclave was subsequently sealed and subjected to a hydrothermal treatment at 120 °C for 8 h. After the reaction had naturally cooled down, the carbon cloth was removed, washed several times with water, and dried at 60 °C under vacuum conditions. Control samples were prepared by adjusting the temperature (90 °C, 150 °C) of the hydrothermal reaction (sample-90 °C-8 h-0.5 M, sample-150 °C-8 h-0.5 M), as well as by varying the reaction time (4 h, 12 h) (sample-120 °C-4 h-0.5 M, sample-120 °C-12 h-0.5 M). Control samples with different NaMoO_4_ solution concentrations (0.1 M, 2 M) were also obtained by adjusting the concentration (sample-120 °C-8 h-0.1 M, sample-120 °C-8 h-2 M).

### 3.3. Synthesis of c-Co/Co_3_Mo on Carbon Cloth

The Co(OH)_2_/CoMoO_4_ precursor (sample-120 °C-8 h-0.5 M) was typically subjected to annealing at 500 °C for 2 h in the central position of a tube furnace. The annealing process was carried out under a flow of Ar/H_2_ gas (100 sccm, 10% H_2_), with a heating rate of 5 °C/min. After the annealing process, the sample was allowed to cool down naturally, resulting in the formation of *c*-Co/Co_3_Mo on the carbon cloth substrate (loading amount ≈ 1.28 mg cm^−2^). To investigate the effect of annealing conditions, the temperature (300 °C, 500 °C) and duration (1 h, 3 h) of the annealing process were adjusted accordingly.

### 3.4. Synthesis of c-Co on Carbon Cloth

To synthesize *c*-Co on carbon cloth, the same annealing conditions as *c*-Co/Co_3_Mo were employed, with the only difference being the replacement of Co(OH)_2_/CoMoO_4_ with α-Co(OH)_2_ as the precursor. The α-Co(OH)_2_ precursor was annealed at the same temperature (500 °C) for the same duration (2 h) in the central position of a tube furnace under an Ar/H_2_ flow (100 sccm, 10% H_2_) with a heating rate of 5 °C/min.

### 3.5. Synthesis of Co_3_O_4_/CoMoO_4_ on Carbon Cloth

For the synthesis of Co_3_O_4_/CoMoO_4_ on carbon cloth, the as-obtained Co(OH)_2_/CoMoO_4_ precursor (sample-120 °C-8 h-0.5 M) was annealed at 500 °C for 2 h in the central position of a tube furnace under an Ar flow. The heating rate during the annealing process was maintained at 5 °C/min.

### 3.6. Preparation of 20% Pt/C Electrode on Carbon Cloth

To prepare the 20% Pt/C electrode on carbon cloth, 10 mg of 20% Pt/C powder was dispersed in a solution containing 100 μL of Nafion solution and 900 μL of anhydrous ethanol. The mixture was subjected to ultrasonic treatment for at least 60 min to ensure the formation of a uniform catalyst ink. The resulting black dispersion was carefully dropped onto the carbon cloth substrate, and the sample was then left to dry overnight in ambient air at room temperature. The loading amount of Pt/C on the carbon cloth was approximately 2.25 mg/cm^2^.

### 3.7. Characterizations

The structures and morphologies of the samples were characterized using various techniques:

#### 3.7.1. Scanning Electron Microscopy (SEM)

The samples were examined using a Hitachi S-4800 field emission scanning electron microscope (Tokyo, Japan), which provided high-resolution images of the surface morphology.

#### 3.7.2. Transmission Electron Microscopy (TEM) with Energy Dispersive X-ray Spectroscopy (EDX)

The JEOL JEM-2100 TEM (Tokyo, Japan) operating at 200 kV was utilized to investigate the internal structures of the samples. EDX analysis was performed simultaneously to obtain elemental composition information.

#### 3.7.3. Powder X-ray Diffraction (XRD)

XRD patterns were obtained using a Bruker D8 ADVANCE instrument equipped with a Cu Kα radiation source (λ = 0.154178 nm). This technique provided information about the crystal structure and phase composition of the samples.

#### 3.7.4. X-ray Photoelectron Spectroscopy (XPS)

Analysis was carried out using a Thermo Fisher ESCALAB XI + XPS instrument (Waltham, MA, USA). This technique allowed for the characterization of the elemental composition and chemical states of the samples.

#### 3.7.5. Raman Spectroscopy

Raman spectra were recorded using a microscopic Raman spectrometer (HORIBA Lab RAM HR Evolution, Kyoto, Japan) with a laser wavelength of 532 nm. Raman spectroscopy provided information about the vibrational modes and molecular structure of the samples.

These characterization techniques collectively provided valuable insights into the structures, morphologies, elemental composition, crystal phases, chemical states, and vibrational properties of the synthesized samples.

### 3.8. Electrochemical Measurements

The electrochemical measurements were conducted using the following setup and procedures: Three-Electrode System: A standard three-electrode system was used, consisting of the prepared samples on carbon cloth as the working electrode, Hg/HgO (1 M KOH) as the reference electrode, and a graphite rod as the counter electrode. Electrolyte: 1 M KOH solution was used as the electrolyte for all measurements. Electrochemical Activation: The electrochemical activation of the working electrode was performed by cyclic voltammetry (CV) in the potential range of 0.124 V to −0.676 V (vs RHE) at a scan rate of 50 mV s^−1^ in 1 M KOH solution. Working Electrode Surface Area: The working surface area of the electrode was controlled to 1 × 0.5 cm for all experiments. Temperature: All tests were conducted at ambient temperature. Potential Conversion: All measured potentials were converted to a reversible hydrogen electrode (RHE) using the equation: E(RHE) = E(Hg/HgO) + 0.059 × pH + 0.098 V. Potential Calibration: All measured potentials were calibrated with iR compensation. Electrochemical Double Layer Capacitance (C_dl_): To determine the electrochemical double layer capacitance (C_dl_), cyclic voltammetry (CV) was performed in the potential range of 0.024 V to 0.074 V (vs. RHE) at various scan rates (20, 40, 60, 80, and 100 mV s^−1^). This potential range was chosen as no apparent Faradaic reactions were observed in this range for all the electrodes. Electrochemical Surface Area (ECSA): The ECSA-normalized current density for the as-prepared catalysts was evaluated using the equation: ECSA-normalized current density = current density × C_s_/C_dl_, where C_s_ is the specific capacitance. The specific capacitance (C_s_) was chosen as C_s_ = 0.040 mF cm^−2^ in 1 M KOH. Turnover Frequency (TOF): The TOF calculation was performed using the equation TOF = j × A/(2 × F × n), where j is the current density, A is the area of the working electrode, F is Faraday’s constant, and n is the number of moles of active sites, assuming that all deposited metals on the electrode are involved in the reaction [56]. Electrochemical Impedance Spectroscopy (EIS): EIS measurements were carried out at −0.1 V (vs. RHE) over a frequency range of 100 kHz to 0.01 Hz with an AC amplitude of 5 mV. Long-Term Stability Tests: Chronopotentiometry (CP) curves were recorded at a current density of 10 mA cm^−2^ to assess the long-term stability of the electrodes. Faraday Efficiency (FE): The Faraday efficiency (FE) of hydrogen production can be determined using the following formula:

FE (%) = (mol of hydrogen)/[(total passed charge)/(2 × F)] × 100%(1)

where F represents Faraday’s constant.

## 4. Conclusions

In summary, this study comprehensively investigated the structural evolution and electrochemical activation of the *c*-Co/Co_3_Mo electrocatalyst during alkaline HER. The instability of the Co_3_Mo alloy and H_0.9_MoO_3_ in the alkaline HER process results in the disruption of the crystal structure, leading to the adsorption of the Mo element and the redeposition and reduction of the Co element. This process gives rise to the formation of coordinated MoO_4_^2−^ ions and *h*-Co, with Co(OH)_2_ serving as an intermediate species. Importantly, these structural changes are accompanied by a significant enhancement in the catalytic activity for alkaline HER. These findings provide valuable insights into the investigation of the structural stability and dynamic catalytic activity of alloy-based electrocatalysts, thus facilitating the rational design of highly efficient catalysts for energy conversion applications.

## Data Availability

Additional data are made available in the Supplementary Materials of this manuscript.

## Supplementary Materials

The following supporting information can be downloaded at: https://www.mdpi.com/article/10.3390/molecules28196986/s1.
