# Supplementary material for: Investigating the Structural Evolution and Catalytic Activity of c-Co/Co3Mo Electrocatalysts for Alkaline Hydrogen Evolution Reaction
Source: Molecules. 2023 Oct 9;28(19):6986. doi: 10.3390/molecules28196986 (PMC10574280; doi:10.3390/molecules28196986)
Supplement: Supplementary file 1 [file molecules-28-06986-s001.zip › molecules-2561202-supplementary.pdf]

Supporting Information

Investigating the Structural Evolution and Catalytic Activity of *c*-Co/Co_3Mo Electrocatalysts for Alkaline Hydrogen Evolution Reaction

Long Chen [1,†], Li-Wen Jiang [1,†] and Jian-Jun Wang [1,2,*]

[1] State Key Laboratory of Crystal Materials, Shandong University, Jinan 250100, China; 202232804@mail.sdu.edu.cn (L.C.); 201812674@mail.sdu.edu.cn (L.-W.J.)
[2] Shenzhen Research Institute, Shandong University, Shenzhen 518057, China
* Correspondence: wangjianjun@sdu.edu.cn
† These authors contributed equally to this work.

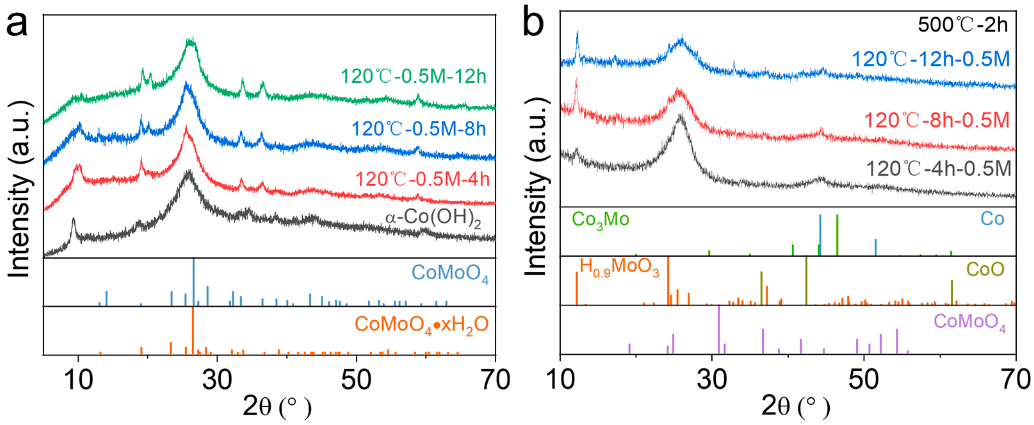

Figure S1. (a) The XRD patterns of sample-120°C-4h-0.5M, sample-120°C-8h-0.5M and sample-120°C-12h-0.5M. (b) The corresponding XRD patterns of catalysts annealed at 500 °C for 2 h in a mixed hydrogen-argon flow.

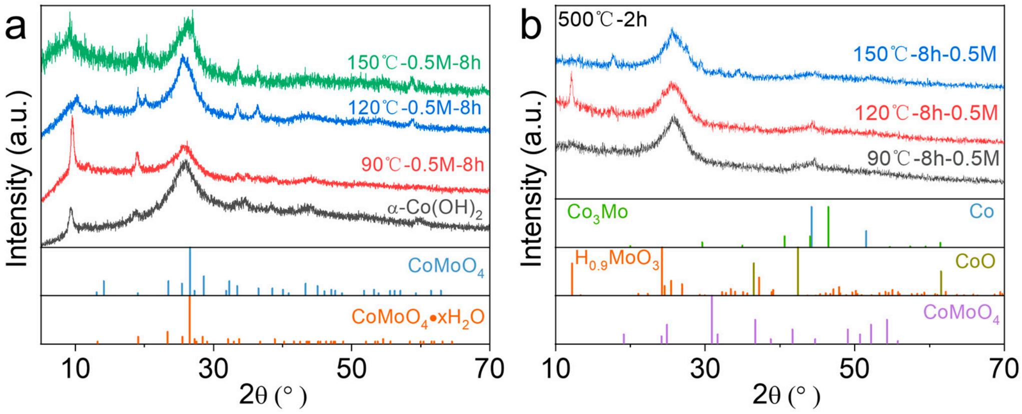

Figure S2. (a) The XRD patterns of sample-90°C-8h-0.5M, sample-120°C-8h-0.5M and sample-150°C-8h-0.5M. (b) The corresponding XRD patterns of catalysts annealed at 500 °C for 2 h in a mixed hydrogen-argon flow.

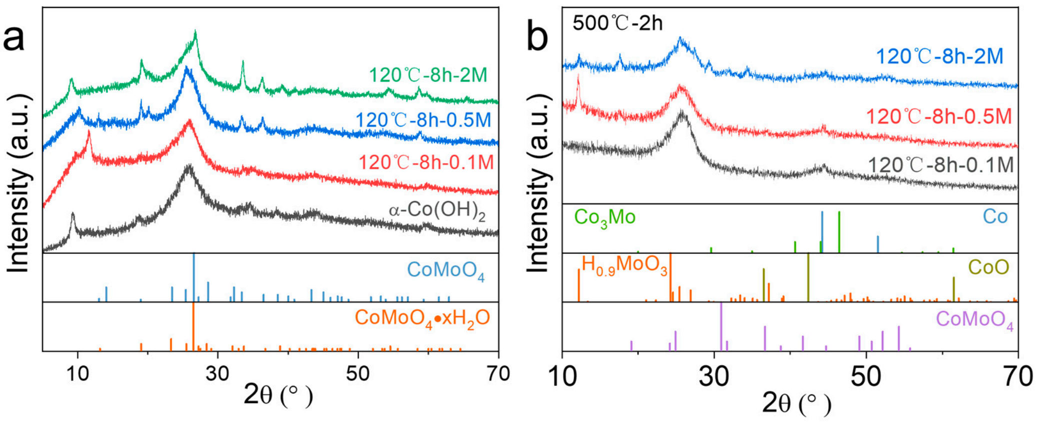

Figure S3. (a) The XRD patterns of sample-120°C-8h-0.1M, sample-120°C-8h-0.5M and sample-120°C-8h-2M. (b) The corresponding XRD patterns of catalysts annealed at 500 °C for 2 h in a mixed hydrogen-argon flow.

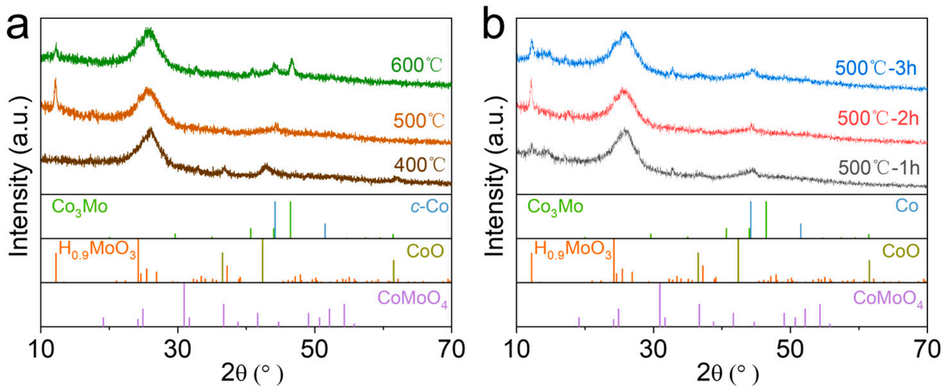

Figure S4. The XRD patterns of *c*-Co/Co_3Mo catalysts obtained by annealing sample-120°C-8h-0.5M at (a) 400°C, 500°C, and 600 °C for 2 h in a mixed hydrogen-argon flow, respectively; (b) 500 °C for 1, 2, and 3h in a mixed hydrogen-argon flow, respectively.

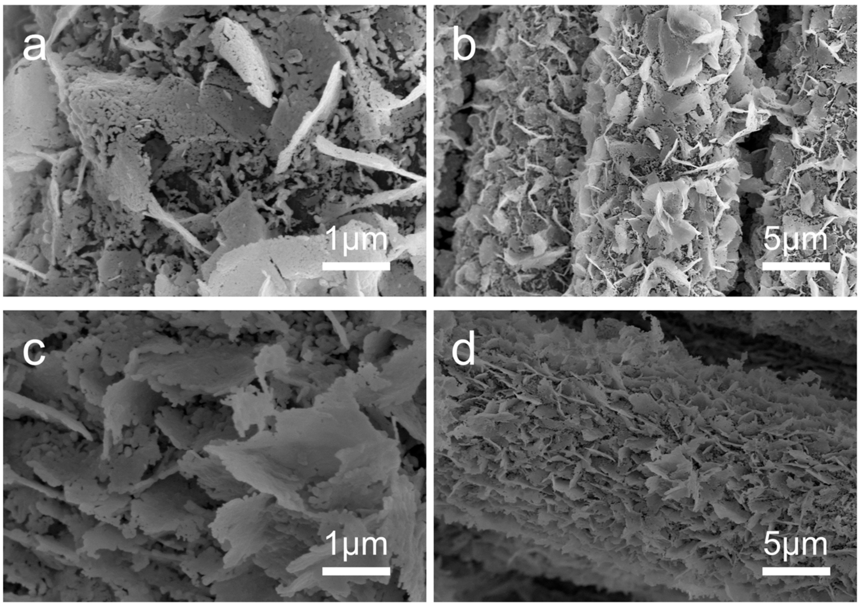

Figure S5. SEM images of catalysts obtained by annealing (a, b) sample-90℃-8h-0.5M and (c, d) sample-150℃-8h-0.5M at 500 °C for 2 h in a mixed hydrogen-argon flow, respectively.

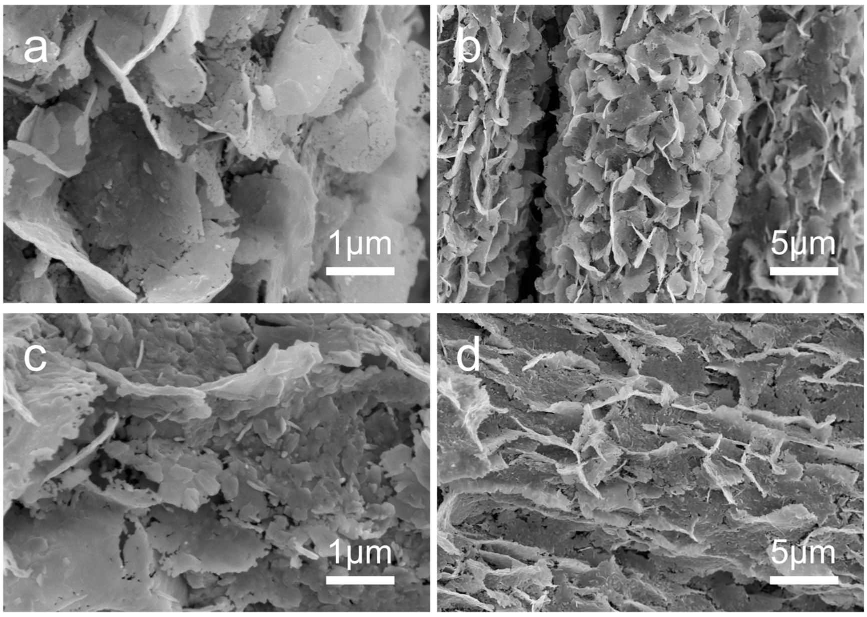

Figure S6. SEM images of catalysts obtained by annealing (a, b) sample-120℃-4h-0.5M and (c, d) sample-120℃-12h-0.5M at 500 °C for 2 h in a mixed hydrogen-argon flow, respectively.

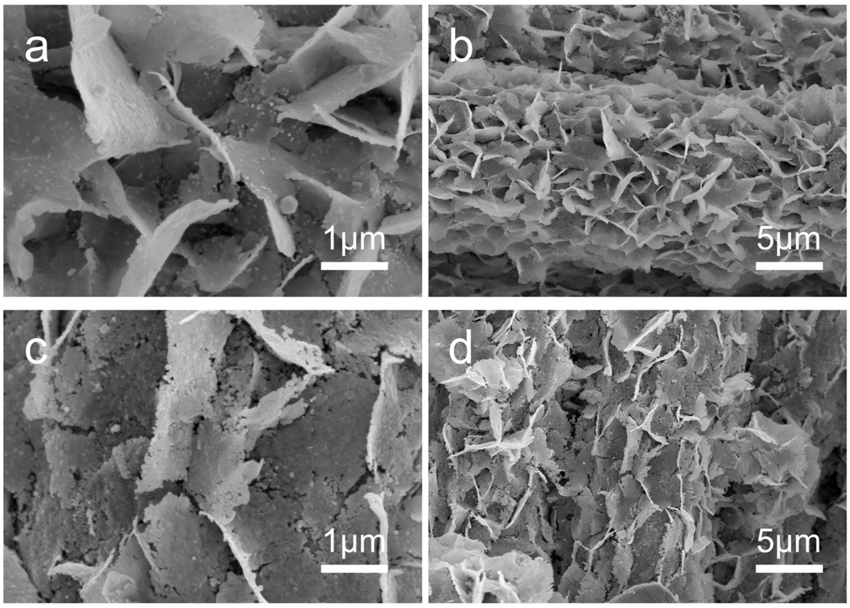

Figure S7. SEM images of catalysts obtained by annealing sample-120°C-8h-0.5M at (a, b) 400 °C and (c, d) 600 °C for 2 h in a mixed hydrogen-argon flow, respectively.

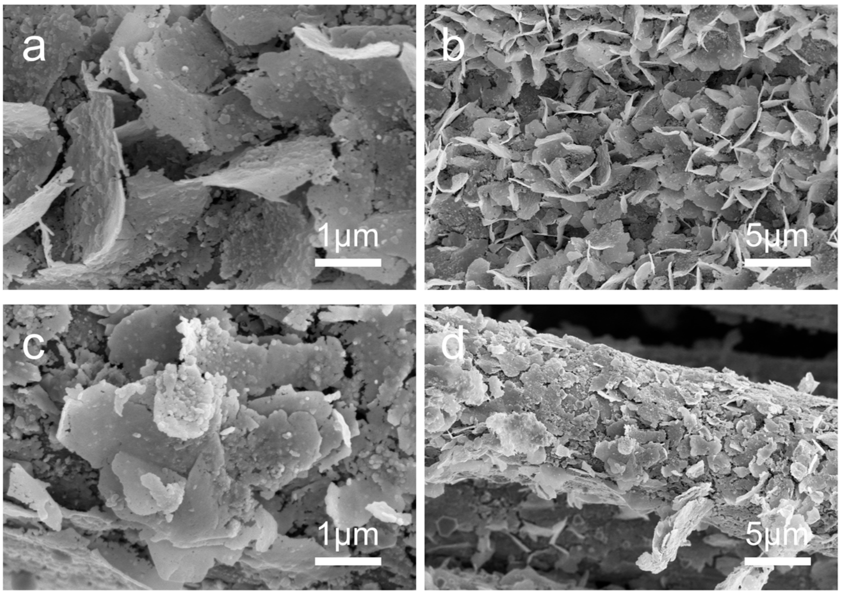

Figure S8. SEM images of catalysts obtained by annealing sample-120°C-8h-0.5M at 500 °C for (a, b) 1 h and (c, d) 3 h in a mixed hydrogen-argon flow, respectively.

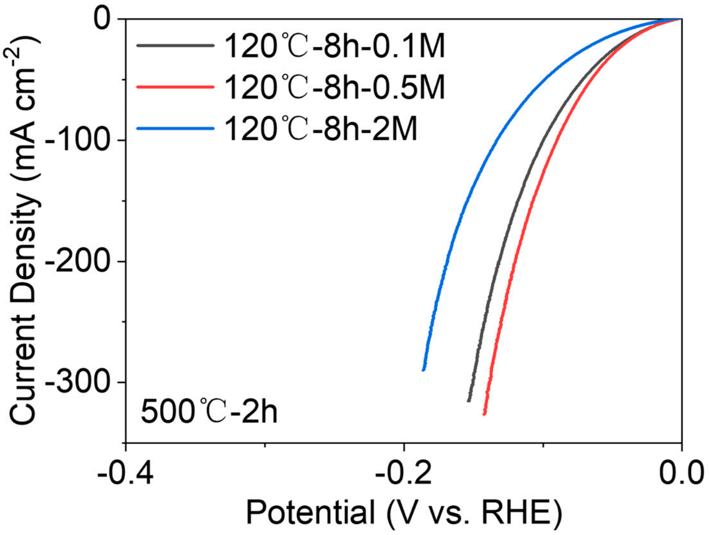

Figure S9. LSV curves of catalysts obtained by annealing sample-120°C-8h-0.1M, sample-120°C-8h-0.5M, and sample-120°C-8h-2M at 500 °C for 2 h in a mixed hydrogen-argon flow, respectively.

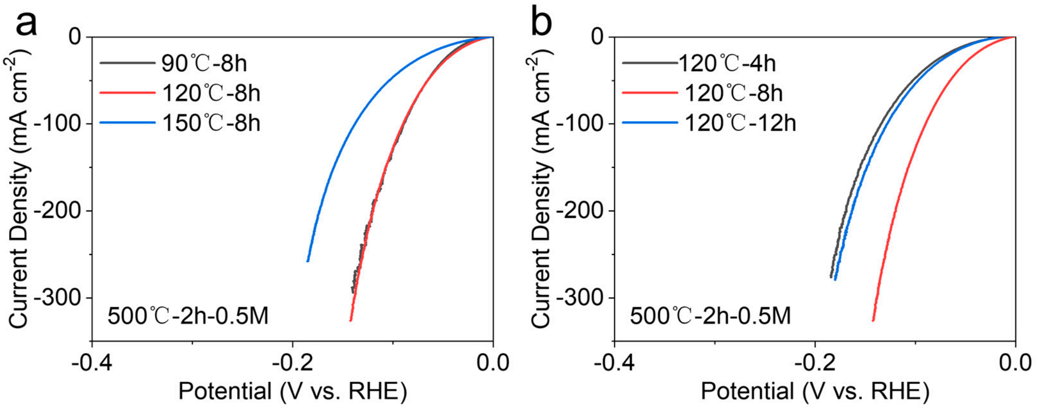

Figure S10. (a) LSV curves of catalysts obtained by annealing sample-90°C-8h-0.5M, sample-120°C-8h-0.5M, and sample-150°C-8h-0.5M at 500 °C for 2 h in a mixed hydrogen-argon flow, respectively. (b) LSV curves of catalysts obtained by annealing sample-120°C-4h-0.5M, sample-120°C-8h-0.5M, and sample-120°C-12h-0.5M at 500 °C for 2 h in a mixed hydrogen-argon flow, respectively.

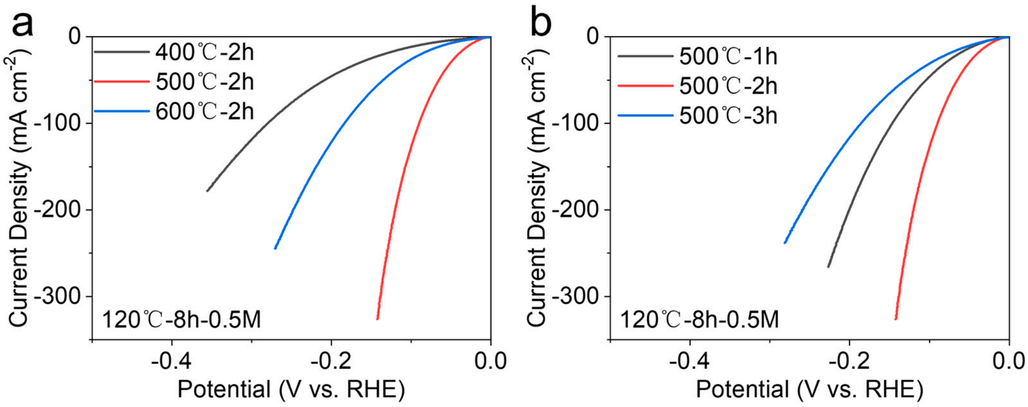

Figure S11. (a) LSV curves of catalysts obtained by annealing sample-120°C-8h-0.5M at 400, 500, and 600 °C for 2 h in a mixed hydrogen-argon flow, respectively. (b) LSV curves of catalysts obtained by annealing sample-120°C-8h-0.5M at 500 °C for 1, 2, and 3 h in a mixed hydrogen-argon flow, respectively.

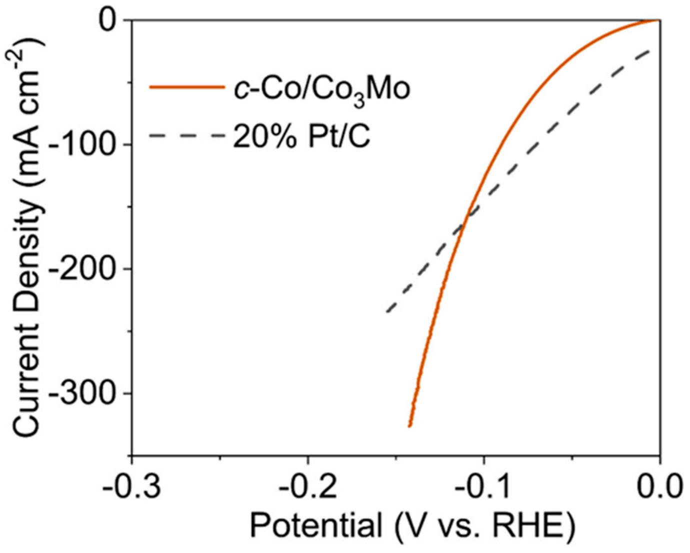

Figure S12. LSV curve of 20% Pt/C and *c*-Co/Co_3Mo electrodes.

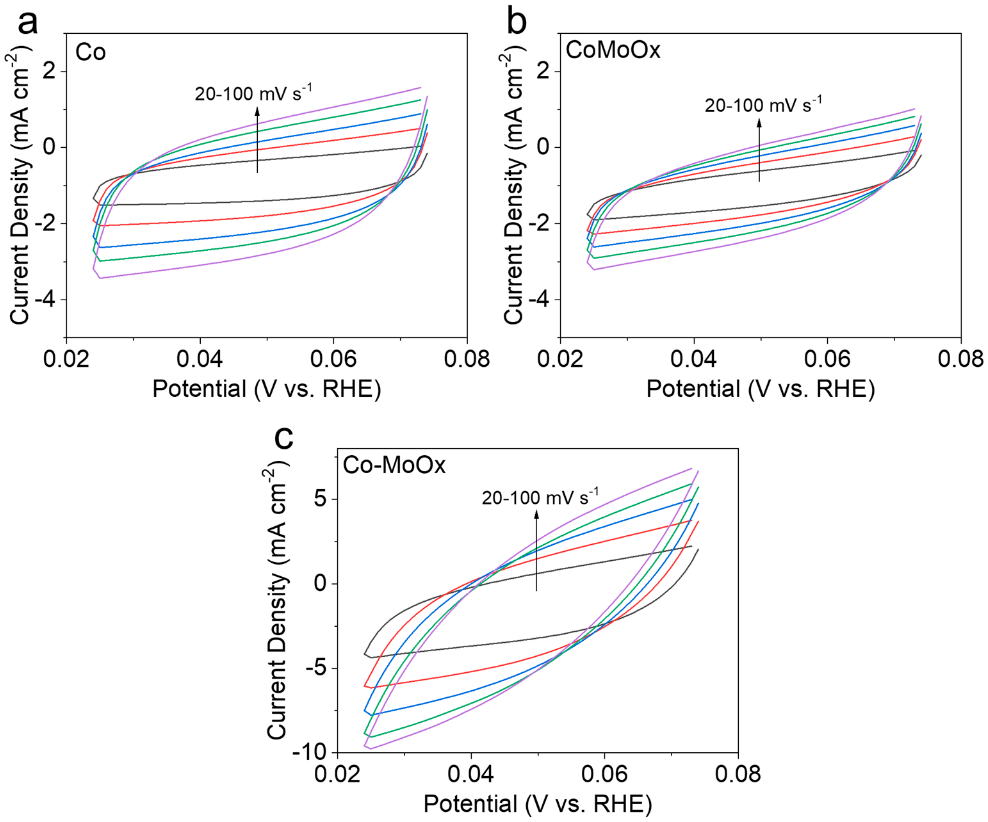

Figure S13. CV curves of (a) *c*-Co, (b) *c*-Co/Co_3Mo, and (c) Co_3O_4/$CoMoO_4$ at varied scan rates.

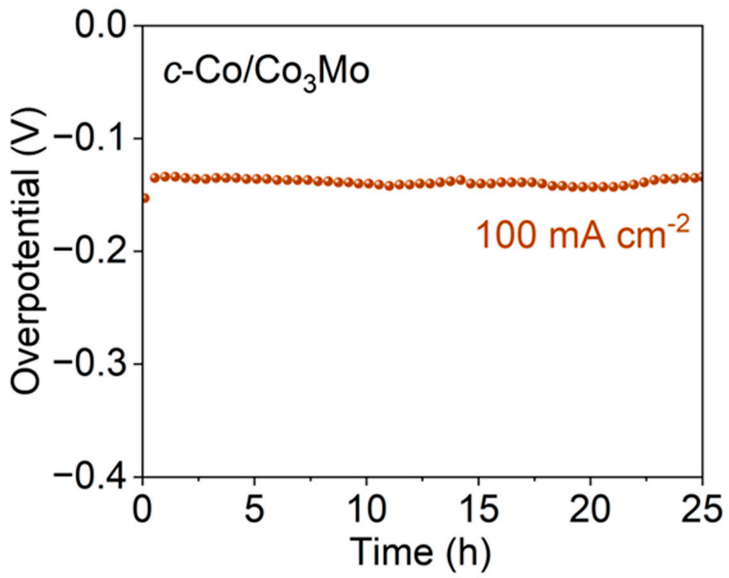

Figure S14. Chronopotentiometry curve of *c*-Co/Co_3Mo at a constant current density of 100 mA cm^{-2}.

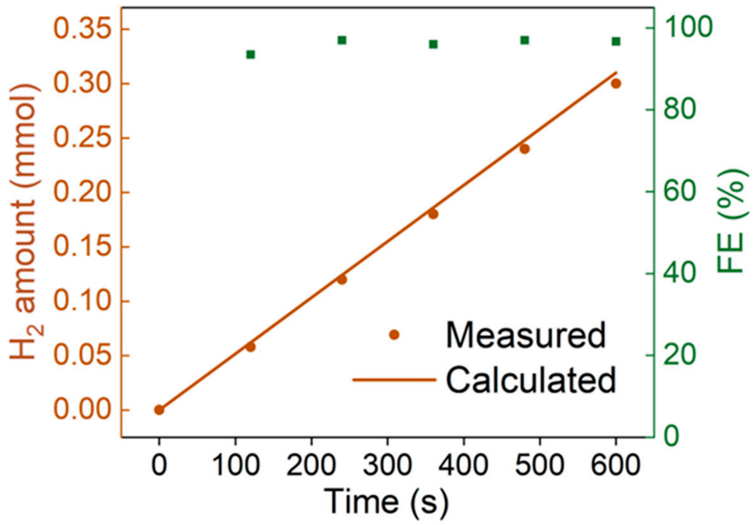

Figure S15. Faraday efficiency of *c*-Co/Co_3Mo at 1 M KOH.

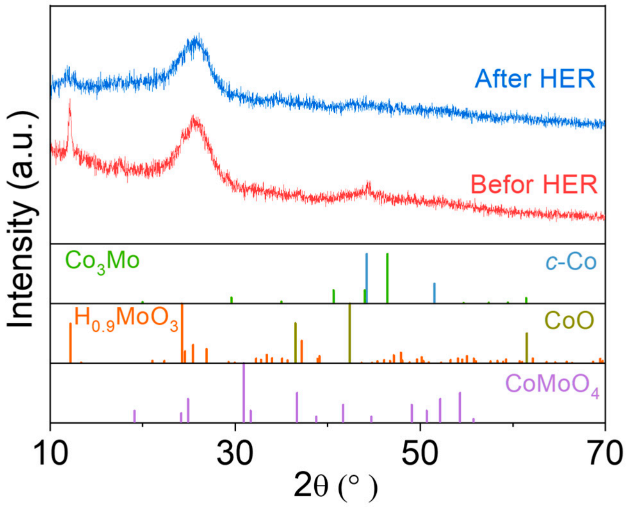

Figure S16. XRD patterns of *c*-Co/Co_3Mo before and after HER test.

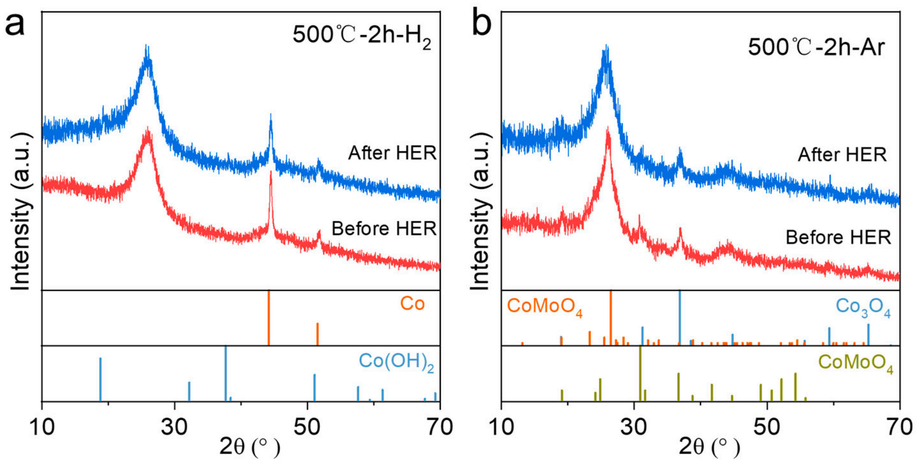

Figure S17. XRD patterns of (a) *c*-Co and (b) $Co_3O_4/CoMoO_4$ before and after HER test.

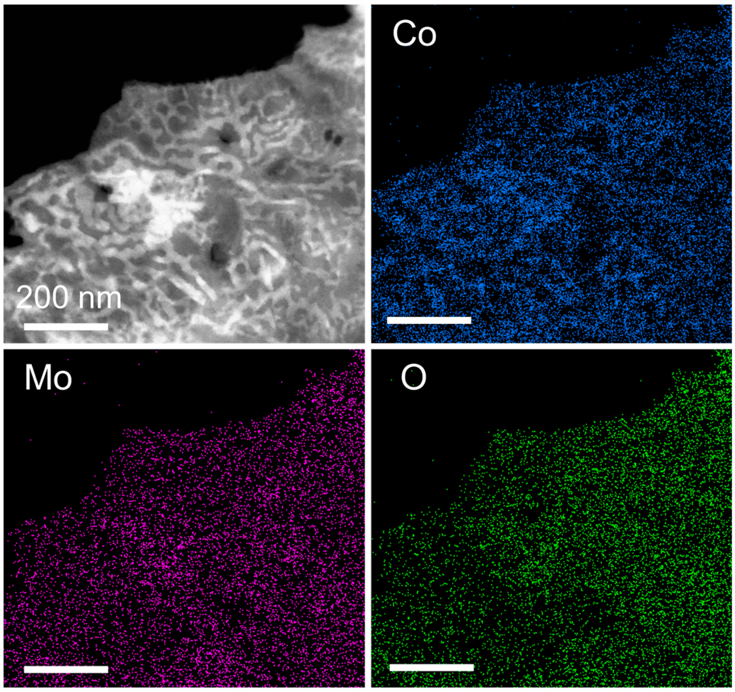

Figure S18. EDX elemental mapping images of *c*-Co/Co_3Mo after HER test.

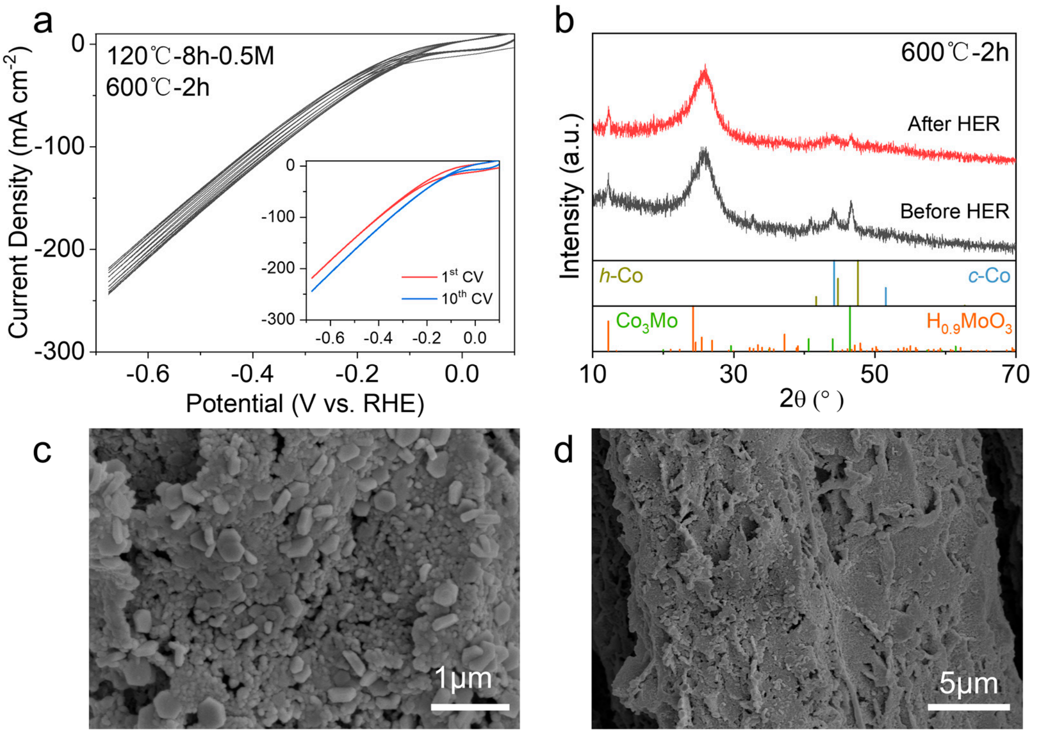

Figure S19. (a) Evolutive CV curves from the 1st to the 10th CV in 1 M KOH at 50 mV s^{-1} between 0.124 and -0.676 V (vs RHE), (b) XRD patterns, (c, d) The SEM image after HER test was obtained when the sample was annealed at 120°C-8h-0.5M, 600°C, in a hydrogen-argon mixed flow for 2 h.

Table S1. The results of EDX for the c-Co/Co_3Mo catalyst.

Element	Wt %	Atomic %
**O**	24.30	56.01
**Co**	61.76	38.64
**Mo**	13.94	5.36

Table S2. The comparison of HER performance for c-Co/Co_3Mo with some related reports.

Catalyst	Overpotential at 10 mA cm^{-2} (mV)	References
***c*-Co/Co_3Mo**	28	**This work**
**RCMO@NF**	68	*ACS Appl. Mater. Interfaces*, **2019**, *11*, 9002– 9010
**Co_3Mo/$CoMoO_3$ NPS**	34	*J. Mater. Sci. Technol.*, **2023**, *137*, 184-192
**NF/CoNiMo-500**	34	*Chem. Eng. J.*, **2022**, *450*, 138206
**PVP- MoS_2-RGO**	66	*Small*, **2018**, *14*, e1803361
**CoP@3DOM-FeP**	67.2	*J. Mater. Chem. A*, **2021**, 9, 23574-23581
**Mn_2P-MnP/PNC**	63	*Chem. Eng. J.*, **2023**, *469*, 143879
**$PdSe_2$**	138	*Adv. Funct. Mater.*, **2021**, *31*, 2102321

Table S3. TOF for *c*-Co/Co_3Mo at overpotentials of 100, 150, and 200 mV for HER.

Overpotential	100 mV	150 mV	200 mV
**TOF s^{-1}**	0.052	0.14	0.24
